# Ultrasonic-Assisted Conversion of Micrometer-Sized BiI_3_ into BiOI Nanoflakes for Photocatalytic Applications

**DOI:** 10.3390/ijms251910265

**Published:** 2024-09-24

**Authors:** Tushar Kanti Das, Marcin Jesionek, Krystian Mistewicz, Bartłomiej Nowacki, Mirosława Kępińska, Maciej Zubko, Marcin Godzierz, Anna Gawron

**Affiliations:** 1 Institute of Physics–Centre for Science and Education, Silesian University of Technology, Krasińskiego 8, 40-019 Katowice, Poland; krystian.mistewicz@polsl.pl (K.M.); miroslawa.kepinska@polsl.pl (M.K.); 2 Department of Industrial Informatics, Faculty of Materials Science, Joint Doctorate School, Silesian University of Technology, Krasinskiego 8, 40-019 Katowice, Poland; bartlomiej.nowacki@polsl.pl; 3 Institute of Materials Engineering, Faculty of Science and Technology, University of Silesia, 75 Pułku Piechoty 1A St., 41-500 Chorzów, Poland; maciej.zubko@us.edu.pl; 4 Department of Physics, Faculty of Science, University of Hradec Králové, Rokitanského 62, 500 03 Hradec Králové, Czech Republic; 5 Centre of Polymer and Carbon Materials, Polish Academy of Sciences, 34. M. Curie-Skłodowskiej St., 41-800 Zabrze, Poland; mgodzierz@cmpw-pan.pl (M.G.); agawron@cmpw-pan.pl (A.G.)

**Keywords:** bismuth triiodide, bismuth oxyiodide, ultrasonic irradiation, methylene blue, photocatalytic

## Abstract

This work describes a novel method for converting bismuth triiodide (BiI_3_) microplates into bismuth oxyiodide (BiOI) nanoflakes under ultrasonic irradiation. To produce BiOI nanoflakes with a high yield and high purity, the conversion process was carefully adjusted. Rapid reaction kinetics and increased mass transfer are benefits of the ultrasonic-assisted approach that result in well-defined converted BiOI nanostructures with superior characteristics. The produced BiOI nanoflakes were examined utilizing a range of analytical methods, such as Transmission Electron Microscopy (TEM), scanning electron microscopy (SEM) and X-ray diffraction (XRD). The progress in the ultrasonic conversion process with time was monitored through diffuse reflectance spectroscopy (DRS). The outcomes demonstrated the effective conversion of BiI_3_ microplates into crystalline, homogeneous, high-surface-area BiOI nanoflakes. Additionally, the degradation of organic dyes (methylene blue) under ultraviolet (UV) light irradiation was used to assess the photocatalytic efficacy of the produced BiOI nanoflakes. Because of their distinct morphology and electrical structure, the BiOI nanoflakes remarkably demonstrated remarkable photocatalytic activity, outperforming traditional photocatalysts. The ability of BiOI nanoflakes to effectively separate and utilize visible light photons makes them a viable option for environmental remediation applications. This work not only shows the promise of BiOI nanoflakes for sustainable photocatalytic applications but also demonstrates a simple and scalable approach to their manufacturing. The knowledge gathered from this work opens up new avenues for investigating ultrasonic-assisted techniques for creating sophisticated nanomaterials with customized characteristics for a range of technological uses.

## 1. Introduction

The unique and fascinating physical properties of crystalline two-dimensional (2D) materials have attracted a lot of attention in research because the electronic states are encased within the van der Waals force of attracted layers [1,2,3]. The intense search for new 2D materials has led to an examination of some layered materials that have really been known for a long time. The popularity of the aforementioned 2D materials encourages scientists to look into new layered ternary compounds that have 2D characteristic properties [4,5]. Bismuth oxyiodide (BiOI) is a common bismuth-based oxyhalide material (known as BiOX, X = F, Cl, Br and I) that has been thoroughly studied for use in photocatalytic wastewater treatment. It is a layered 2D material that is a build-up of easily exfoliated stacked layers of [I-Bi-O-Bi-I] units linked through the van der Waals force of attraction [6,7,8]. BiOI has a tetragonal structure with lattice constants of a = b = 0.3994 nm and c = 0.9149 nm. It has a 2D layered structure, with [Bi_2_O_2_] slabs interspersed with double slabs of iodine atoms in the tetragonal matlockite structure. The substantial interspace between the layers can polarize atoms, which effectively separates the hole–electron (e^−^/h^+^) pair and facilitates photocatalytic process [9,10]. In addition, BiOI’s extensive spectrum of visible spectra compels us to examine it as a material for absorption or a potential photocatalytic material for solar applications [11]. Also, the indirect transition band gap of BiOI needs the excited electron to traverse a specific k-space distance for release, which minimizes the possibility of hole–electron (e^−^/h^+^) pair recombination. Due to this fact, BiOI has been considered as a highly effective photocatalytic material under light irradiation [9,12]. As a consequence, the research community is developing nanostructured BiOI through numerous methods. Among those methods, hydrothermal, solvothermal, precipitation, reverse microemulsion, electrodeposition, microwave and template are commonly employed methods for the preparation of different morphology-based nanostructured BiOI materials [13]. Selecting the right synthesis protocol for the large-scale production of nanomaterials is crucial for achieving economic viability. The synthesis of 2D nanoscale materials like graphene follows a top-down approach for large-scale production, while the preparation of 2D BiOI nanomaterials are pursued with bottom-up methods, as this type of 2D nanomaterial is formed by the binding and interaction of Bi-O-I bonds [14].

Recently, the creation of nanoscale inorganic materials through the ultrasonic irradiation method has become a valuable tool. Also, the study of the physical and chemical effects of the ultrasonic radiation method is a rapidly expanding field of research [15,16]. Microbubbles in liquid can be created by ultrasonic irradiation. Their cavitational collapses result in powerful shock waves, liquid jet streams traveling at approximately 400 km/h, heating and cooling rates exceeding 10^10^ K/s and temperatures as high as 5000 K and 1000 atm [17,18]. The sonochemical method offers several advantages for material conversion. It enhances reaction rates due to the localized high temperatures and pressures generated by ultrasonic waves, leading to faster synthesis. This method often results in materials with improved properties, such as smaller particle sizes, higher surface areas and better catalytic activity. The intense mixing from ultrasonic waves also ensures uniform particle distribution and reduces agglomeration. Additionally, it is energy-efficient and aligns with green chemistry principles by operating under milder conditions and using fewer chemicals. The method’s versatility allows it to be applied to a wide range of materials and its scalability makes it suitable for both laboratory and industrial applications [19,20]. In contrast to the typical methods for synthesizing nanoscale materials, earlier investigations showed that such an extreme chemical and physical environment was useful to improve the rate of synthetic processes and to obtain various crystalline materials [21,22,23]. Until now, this ultrasonic method has not been applied for synthesizing nanostructured BiOI through the conversion of bismuth-based iodide materials.

In this paper, ultrasound sonochemistry was used for the conversion of synthesized hexagonal bismuth triiodide (BiI_3_) microplates into BiOI nanoflakes using ethanol as a reaction medium. The progress in the conversion process and ultimate conversion into BiOI nanoflakes was continuously kept under observation by diffuse reflectance spectroscopy (DRS). The synthesized BiI_3_ microplates and ultrasonically converted BiOI nanoflakes were well characterized by electron microscopic techniques. The crystal structures and elemental identification of both synthesized BiI_3_ and converted BiOI were confirmed by the X-ray diffraction (XRD) method and energy-dispersive X-ray spectroscopy (EDS), respectively. Finally, a thorough investigation was conducted into the relationship between the BiOI as a photocatalyst’s structure and properties. To assess the photocatalytic activity of BiOI materials, methylene blue (MB) photodegradation was used. Additionally, a plausible mechanism for the photodegradation of pollutants in this system was suggested, grounded in tests involving the trapping of free radicals. The work will be useful for the rapid preparation of BiOI nanoflakes and degrading dyes in the future, which will regulate and reduce environmental pollution. 

## 2. Results and Discussion 

Innovative methods in chemical synthesis continue to expand the frontiers of material science. In this pursuit, BiOI emerges as a promising candidate, synthesized via the rapid and facile ultrasonic treatment of BiI_3_ in an ethanol medium. This novel approach not only underscores the versatility of ultrasonic techniques but also offers a pathway to tailor unique properties in bismuth-based oxyhalide (BiOX) material. 

### 2.1. Characterization of Synthesized Bismuth Triiodied (BiI_3_) Microplates and Ultrasonically Converted Bismuth Oxyiodide (BiOI) Nanoflakes

**Figure 1:**
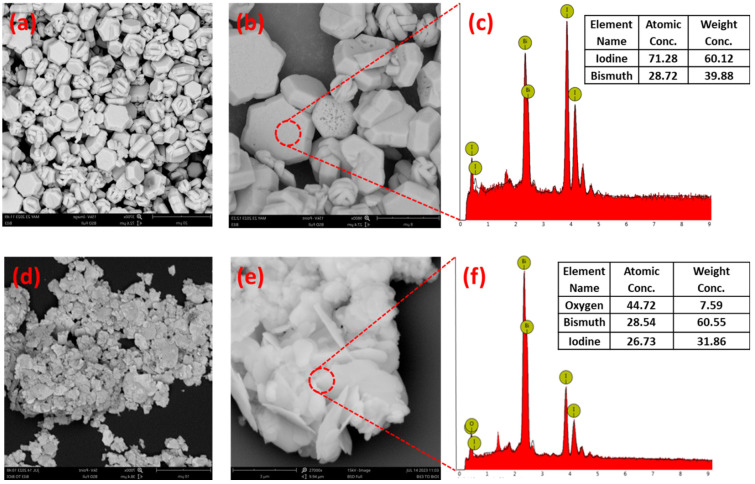
SEM images of (**a**) BiI_3_ microplates (Scale Bar: 20 μm), (**b**) the magnified view on the surface of BiI_3_ microplates (Scale Bar: 8 μm) and (**c**) the EDS pattern of BiI_3_ microplates (inset: elemental composition). SEM images of (**d**) BiOI nanoflakes (Scale Bar: 10 μm), (**e**) the magnified view of BiOI nanoflakes (Scale Bar: 3 μm) and (**f**) the EDS pattern of BiOI nanoflakes (inset: elemental composition).

The morphology and qualitative elemental analysis of the synthesized BiI_3_ and ultrasonically converted BiOI product were explored in depth through SEM analysis and the results of these investigations have been depicted in Figure 1. The single hexagon microplate structure of the synthesized BiI_3_ is clearly shown in Figure 1a. The magnified image (Figure 1b) revealed well-grown hexagonal sheets having smooth surface with precise corners. The thicknesses of hexagonal microplates are varied from 0.8 μm to 3.0 μm, having an average ~1.68 μm (as presented in Figure S1). The EDS analysis (as presented in Figure 1c) demonstrates the presence of bismuth and iodine as elements without any other impurities and the atomic concentration of these elements is in good agreement with previously published results [24]. After 10 min ultrasonic treatment of BiI_3_, the hexagon microplate-like morphology is converted into nanoflakes due to the formation of BiOI (Figure 1d). The magnified image (Figure 1e) shows the large number of smooth and sharp-edge BiOI nanoflakes aggregated into a flower-like structure. The measured thicknesses of the nanoflakes are in the range of 30 nm to 100 nm with an average of ~50 nm (Figure S2). The EDS analysis of the converted BiOI (as presented in Figure 1f) affirms the presence of bismuth, oxygen and iodine, which suggest the successful conversion of BiI_3_ microplates into nanoflakes of BiOI through the sonochemical process [25].

**Figure 2:**
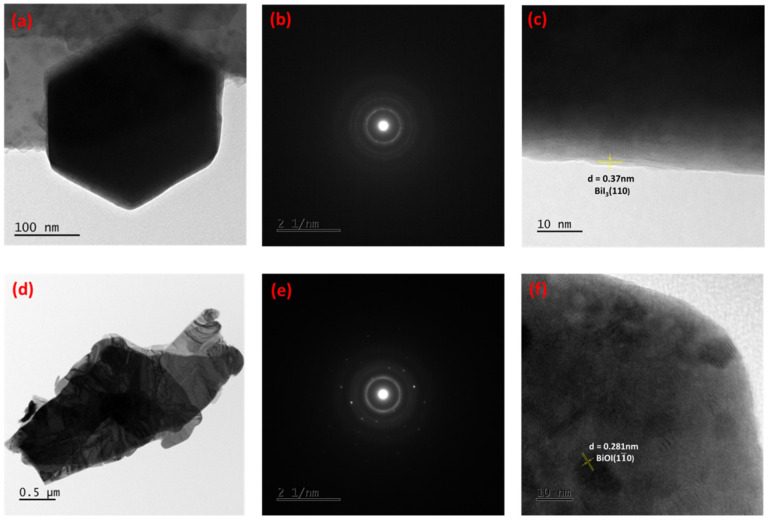
(**a**) TEM image, (**b**) SAED pattern and (**c**) lattice fringe measurement of BiI_3_ (d is the spacing of the (110) planes). (**d**) TEM image, (**e**) SAED pattern and (**f**) lattice fringe measurement of BiOI (d is the spacing of the (1$\bar{1}$0) planes).

To obtain nanoscale resolution and the ability to examine interior structures, TEM analysis has been also performed on both synthesized and converted products. The results of this examination are demonstrated in Figure 2. TEM image of the single hexagon BiI_3_ microplates having a length of around ~200 nm (distance from one corner to another) is presented in Figure 2a. The analogous selected area electron diffraction (SAED) patterns in Figure 2b demonstrate a high degree of crystallinity, as noticed from the diffraction spot pattern. The good-crystalline-quality BiI_3_ is also evidenced by the presence of lattice fringes, as demonstrated in Figure 2c. The measured spacing between the lattice fringes is 0.37 nm, corresponding to the (110) plane of rhombohedral BiI_3_ crystal (Figure 2c), which is identical to previously published results [26]. The result of lattice fringe measurement is demonstrated in Figure S3. After the ultrasonic treatment of BiI_3_, the microplate-like morphology is converted into thin nanoflakes of BiOI, as evidenced by Figure 2d. The figures show that BiOI consists of nanoflakes that overlap each other. This is consistent with the SEM results. The corresponding SAED pattern (presented in Figure 2e) demonstrates clear diffraction spots, indicating the single-crystalline nature of BiOI. The calculated lattice fringe of BiOI is 0.281 nm (Figure 2f). This can be attributed to the (1$\bar{1}$0) plane of the tetragonal BiOI crystal, which is consistent with previously published findings [27]. The result of measuring lattice fringe is presented in Figure S4.

**Figure 3:**
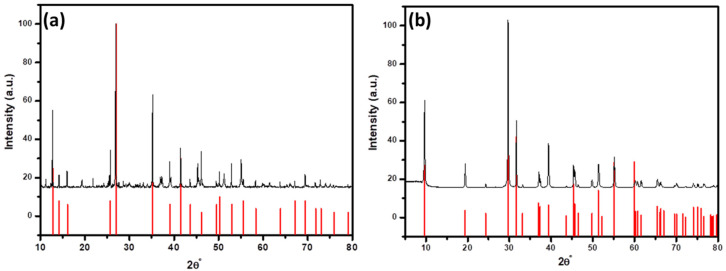
XRD pattern of (**a**) BiI_3_ microplate and (**b**) ultrasonically converted BiOI nanoflakes. (Red lines represent standard JCPDS data and black lines describe the experimental data obtained from the synthesized materials).

The phase purity and crystalline structure of synthesized BiI_3_ and ultrasonically converted BiOI were examined through XRD and the results are demonstrated in Figure 3. Figure 3a shows the XRD pattern of the as-prepared BiI_3_ microplates, which are phase-pure rhombohedral BiI_3_ (PCPDF-48-1795) with no other impurities [28]. The XRD-pattern-converted BiOI nanoflakes (as presented in Figure 3b) are identical to tetragonal BiOI (PDF 04-012-5693), with no evidence of BiI_3_, suggesting complete conversion [29]. The results further indicate that the synthesized rhombohedral BiI_3_ interacts with ethanol under ultrasonic irradiation, primarily forming tetragonal BiOI within 10 min. In addition, the average crystallite size was determined by broadening the highly intense XRD peak [based on the (113) reflection plane of BiI_3_ and the (102) reflection plane of BiOI] using Scherer’s formula in Equation (1) [30]:

(1) $$L=\frac{K\lambda }{\beta \cos\theta }$$

where *L* = crystallite size, *K* is the Scherrer constant (0.9), *λ* = wavelength of the X-ray beam used (0.154 nm), β = Full width at half maximum (FWHM) of the peak and *θ* = Bragg angle. It was found that BiI_3_ and BiOI have average crystallite sizes of 111.12 nm and 44.19 nm, respectively.

**Figure 4:**
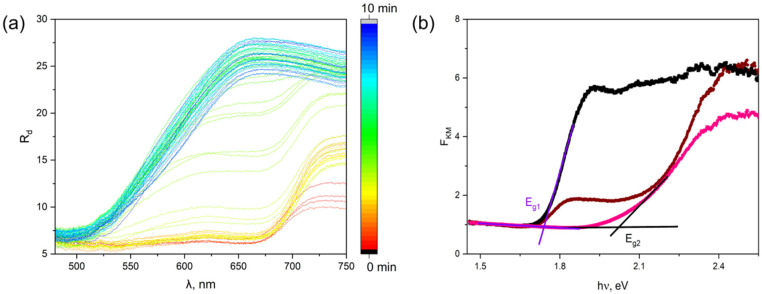
Change in the diffuse reflectance spectrum during synthesis (**a**), the Kubelka–Munk function (**b**) of BiI3 (■) (t = 0 min), the intermediate phase (●) (t = 5 min) and BiOI nanoflakes (▲) (t = 10 min); black and red straight lines represent fitted linear approximations for the optical energy gap calculation. The detailed description is provided in text.

DRS spectra were recorded every 5 s during synthesis. The results are shown in Figure 4a. The process of transition of BiI_3_ to BiOI can be observed. The process is sudden. However, it can be observed that the actual break takes place at the very end of the synthesis process. This may indicate the formation of core–shell material (BiI_3_-BiOI) in the first stage of synthesis. Only in the final stage of conversion, BiI_3_ undergoes full conversion to BiOI. The quantified DRS attributes enable the computation of the Kubelka–Munk function (*F_KM_*) (as illustrated in Figure 4b) through the prescribed Equation (2):

(2) $$F_{KM}=\frac{{\left(1-R_{d}\right)}^{2}}{2R_{d}}\sim \alpha$$

where *R_d_* is diffusive reflectance and *α* is the absorption coefficient of light in the investigated material. The Kubelka–Munk functions were utilized for determining the optical energy gap of BiI_3_ (E_g1_) and BiOI Nanoflakes (E_g2_). Detailed descriptions of different techniques for determining the optical energy gap are discussed in [31].

By examining the Kubelka–Munk function of BiI_3_ and BiOI nanoflakes (Figure 4), it becomes evident that there are two linear segments with fitted lines for each curve. Employing method number 5 outlined in the literature [31], the optical energy gap for BiI_3_ and BiOI nanoflakes can be ascertained as the point where the extrapolations of the straight lines intersect, below and above the small-photon energy knee. During the conversion process of BiI_3_ to BiOI, the spectra for intermediate states were recorded. This can be observed in Figure 4a, where a small number of transient spectra illustrate the rapid avalanche course of the synthesis reaction. One of the spectra is shown in Figure 4b. It consists of parts characteristic of the starting and ending compounds.

Hence, the energy gaps computed for BiI_3_ and BiOI nanoflakes are E_g1_ = 1.743(33) eV and E_g2_ = 2.022(65) eV, correspondingly. The obtained optical energy band gap values for BiI_3_ and BiOI nanoflakes are in line with those reported for BiI_3_ (E_g_ = 1.74 eV) [32] and BiOI (E_g_ = 1.94 eV) [33].

### 2.2. Mechanism behind Conversion of BiI_3_ Microplates into BiOI Nanoflakes

**Figure 5:**
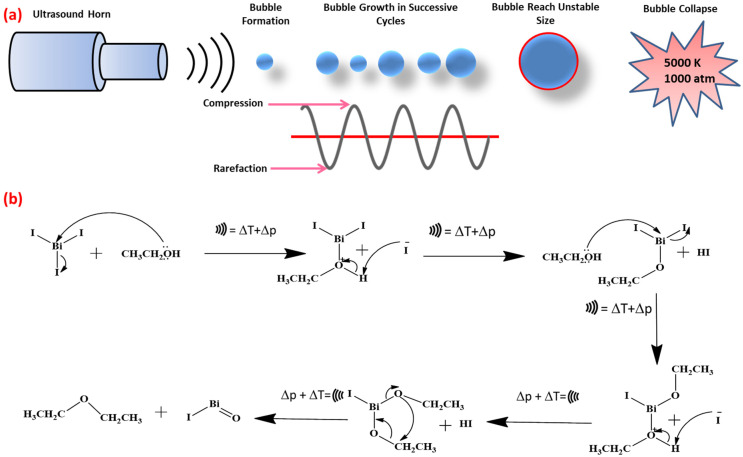
(**a**) Bubble formation and collapse in acoustic cavitation during the ultrasonic process and (**b**) the conversion mechanism of BiI3 into BiOI under ultrasonic irradiation in ethanol medium (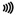 represents ultrasonic wave and ∆p and ∆T denotes the change in pressure and temperature respectively).

The plausible conversion mechanism of BiI_3_ into BiOI in ethanol medium under ultrasonic irradiation is schematically presented in Figure 5. The reaction can take place only in the presence of ethanol but it will take a longer time for conversion. The presence of ultrasonic irradiation can reduce the conversion time to 10 min. The reason behind the reduction in the conversion time is that ultrasonic irradiation in an ethanol medium produces a large amount of heat and pressure through the formation of the bubble and its collapsing [34] (as presented in Figure 5a), which accelerates each step of the reactions. The average bubble size formed during acoustic cavitation under ultrasonic irradiation can vary depending on the ultrasonic frequency, power and the properties of the medium (e.g. viscosity and surface tension). In our case, the ultrasonic bath operates at a frequency of 20 kHz, which typically generates bubbles in the range of 15–150 µm in diameter. While exact measurements of bubble size were not taken in our study, it is well established in the literature that the bubble size in this frequency generally falls within this range [35,36]. This range of bubble size is important for efficient cavitation and the subsequent collapse of bubbles, which generate localized high temperatures and pressures, aiding in the transformation of BiI_3_ into BiOI. The steps of the conversion through the formation of various intermediates are demonstrated in Figure 5b. In the first step of the reaction, the lone pair of oxygen atoms undergo a nucleophile substitution reaction with a vacant p-orbital of Bi atoms followed by the release of I atoms from BiI_3_ through the release of hydroiodic acid (HI) as a side product. Similar steps take place in the following next two steps until another I atom is released from the intermediate compound and forms a bismuth iodide alkoxide [IBi(OCH_2_CH_3_)_2_]-type intermediate. In the last step of the reaction, this unstable intermediate undergoes intramolecular cyclization reaction in the presence of high heat and pressure (i.e. it is produced from the collapsing of bubbles from ultrasonication in the ethanol medium) to produce bismuth oxyiodide (BiOI) and diethylether (CH_3_CH_2_-O-CH_2_CH_3_) as side products.

### 2.3. Photocatalysis 

#### Photocatalytic Degradation of Methylene Blue (MB)

Methylene blue (MB) is a water-soluble cationic dye, which can produce serotonin overdose and severe central nervous system toxicity. At room temperature, the dye appears as a deep blue, less solid substance. The textile industry releases this harmful dye into water bodies, where it can burn human eyes or perhaps cause irreversible damage to aquatic species’ eyes [37]. Based on the above background we selected model dye pollutants to examine the photocatalytic activity of converted BiOI nanostructured material under UV-visible light illumination.

**Figure 6:**
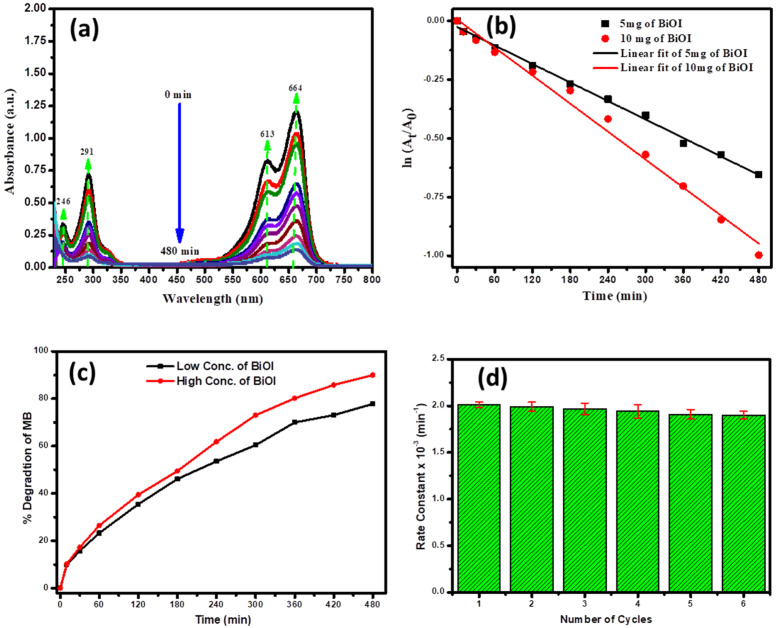
(**a**) Representative time-dependent absorption spectra of the photocatalytic degradation of the MB solution; (**b**) plot of ln(A_t_/A_0_) vs. time for different amounts of BiOI photocatalyst toward the degradation of MB solution (■—5 mg and ●—10 mg). The black and red lines present the best-fitted line according to pseudo-first-order kinetics for 5 mg and 10 mg, respectively. (**c**) The photodegradation efficiency (%) toward MB solution at different time intervals for various amounts of BiOI photocatalyst (the black and red lines represent 5 mg and 10 mg of the BiOI sample, respectively). (**d**) Degradation rate constant of MB by BiOI for six experimental runs (10 mg sample).

The results of BiOI nanoflakes’ photocatalytic activity towards the degradation of MB in the aqueous solution under an ultraviolet visible lamp are presented in Figure 6. The representative time-dependent degradation spectrum of MB is presented in Figure 6a. The figure clearly shows the characteristic UV-visible absorption peaks at 664, 613, 291 and 246 nm of MB, which are reduced with time and reach almost 90% reduction within 8 h in the presence of UV-light and the BiOI sample (10 mg). For this study, we used a very small amount (5 mg and 10 mg) of sample to avoid the effect of adsorption of MB by the sample. Based on the decreased characteristic UV-visible peak at 664 nm of MB, we evaluated the rate of degradation using following 1st-order kinetic Equation (3) [38,39]:

(3) $$\ln\frac{A_{t}}{A_{0}}=-\mathrm{kt}$$

where A_t_ and A_0_ are of the absorbance at a specific time t and t = 0, respectively; k is the degradation rate constant (in min^−1^); and t is time (in min).

To evaluate the degradation rate constant (k), we constructed a plot of ln(A_t_/A_0_) vs. time (t) based on the experimental data (as presented in Figure 6b) and from the slope of the linearly fitted plot, we measured the rate constant 1.321 × 10^−3^ and 2.01 × 10^−3^ min^−1^ for 5 mg and 10 mg of the BiOI sample, respectively. The value of regression coefficients (r^2^) is above ≅0.99, which suggests good agreement of the fitted plot with the experimental data. In addition, the degradation efficiency (*η*) was determined using the following Equation (4) [40]:

(4) $$\eta =\frac{C_{0}-C_{t}}{C_{0}}\;100$$

where *C*_0_ is the initial concentration of MB and *C_t_* denotes MB concentration after t time of ultraviolet visible lamp irradiation. Here, we observe that with increasing minutes of the BiOI sample, the % MB degradation is increased for a specific time (as presented in Figure 6c). We can degrade almost 90% of MB within 8 h in the presence of only a 10 mg BiOI sample, while it is 79 % with 5 mg of BiOI. In addition, the reusability of the BiOI photocatalyst was assessed by performing multiple cycles of MB dye degradation under identical experimental conditions. Figure 6d shows the photocatalytic performance of BiOI up to the sixth cycle. The results demonstrate that the BiOI nanoflakes retain a high degradation rate after the sixth cycle, with only a slight decrease in the MB degradation rate. After the sixth consecutive cycle, the degradation efficiency decreased only by approximately 5.5%, indicating good stability and recyclability of the BiOI nanoflakes. These findings confirm the robustness of the prepared BiOI nanoflakes and their photocatalytic performance remains effective over multiple uses. The slight reduction in efficiency can be attributed to minor losses of catalyst material during recovery or possible surface deactivation, but overall, the results show that BiOI nanoflakes are a stable and reusable photocatalyst for MB degradation This suggests that the BiOI photocatalyst retains a substantial portion of its activity even after multiple uses, making it a viable candidate for repeated applications in wastewater treatment. The measured % of degradation and the time of the reaction towards the photocatalytic degradation of MB is also compared with the previously published results and the comparison of the rate constant is summarized in the Table 1. The table shows that the converted BiOI nanoflakes demonstrated either comparable or higher photocatalytic activity towards degradation of MB in comparison to previously employed various nanomaterials and composite materials. 

### 2.4. Photocatalytic Degradation Mechanism of Methylene Blue (MB) 

**Figure 7:**
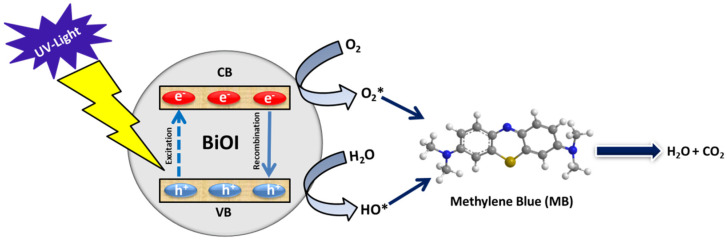
Schematic mechanism of photocatalytic degradation of MB in the presence of BiOI as a photocatalyst.

**Figure 8:**
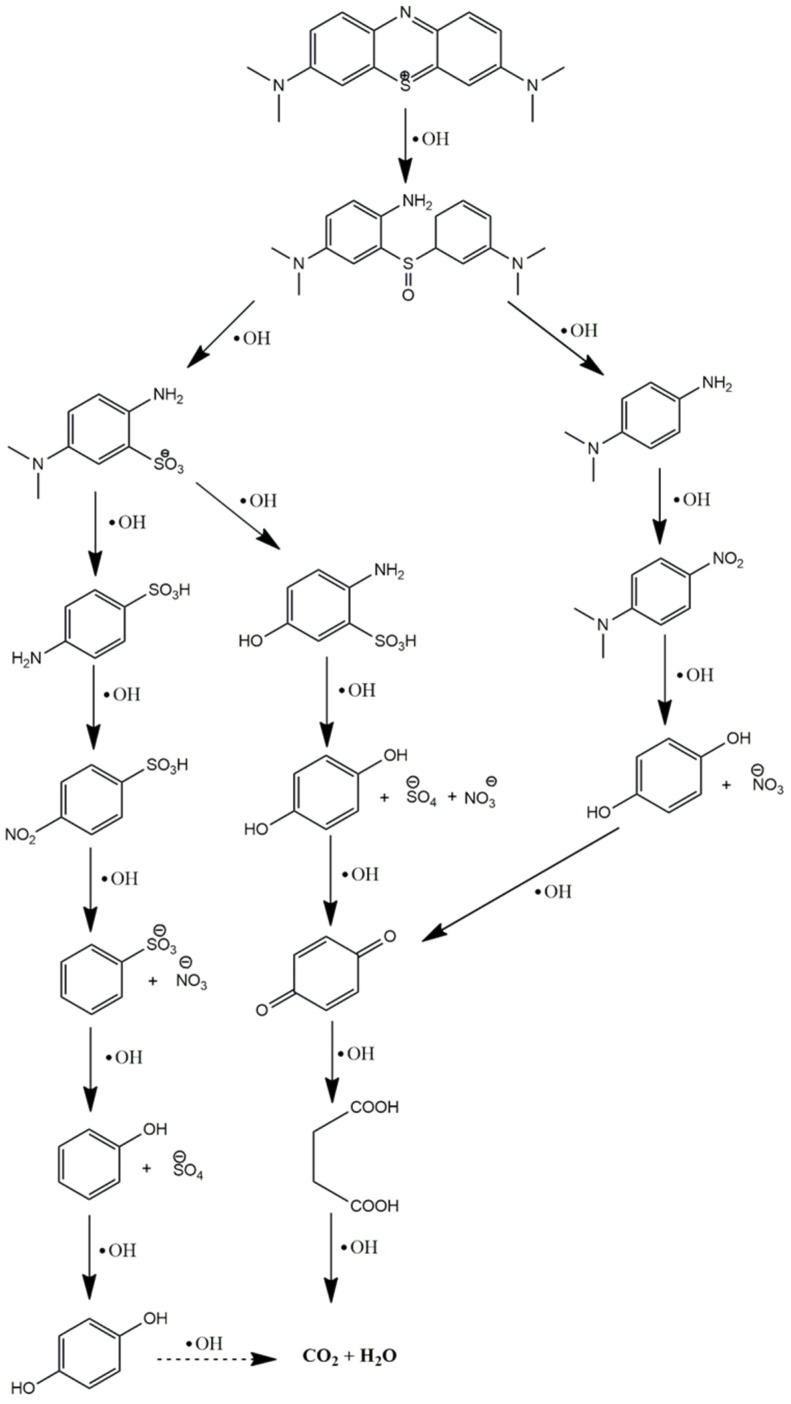
Stepwise degradation pathway of the photocatalytic oxidation of MB molecules.

The photocatalytic degradation of pollutants conducted in the presence of either ultraviolet (UV) light or visible light by photocatalytic active materials is able to produce highly active radicals, which are responsible for the degradation of toxic pollutants such as MB present in the aqueous medium. The complete procedure can be broken down into several steps, as shown in Figure 7: (1) the creation of electron–hole (e^−^/h^+^) pairs causes light to be absorbed, activating the photocatalyst and these react with the water and oxygen in the aqueous medium to create highly reactive oxygen species like superoxide ions (O_2_^−^) and hydroxyl radicals (·OH); (2) the MB molecules gradually diffuses from the aqueous phase to the surface of the photocatalyst; (3) later, MB molecules are adsorbed on the surface of the photocatalyst; (4) the photocatalytic reaction between the adsorbed MB molecules and highly active radical species occurs; and (5) the degraded materials of the photocatalytic reaction are diffused away from the surface of the photocatalyst and the catalyst is ready for the next cycle [49]. Both superoxide ions (O_2_) and hydroxyl radicals (·OH) are responsible for the degradation of MB molecules through the photo-oxidation and photo-reduction processes, respectively. The degradation pathways of MB molecules are illustrated in Figure 8. During the photocatalytic oxidation process, hydroxyl radicals initially target the N-S heterocyclic group due to the higher electron density of the sulfhydryl group. This leads to the breakdown of methylene blue into 2-amino-5-dimethylaminobenzenesulfonic acid anion and dimethyl-(4-nitrophenyl) amine. The latter is then arrested by hydroxyl radicals, transforming into p-dihydroxybenzene. Meanwhile, 2-amino-5-dimethylaminobenzenesulfonic acid anion is further degraded into 4-aminobenzenesulfonic acid and 2-amino-5-dimethylaminobenzenesulfonic acid. The 4-aminobenzenesulfonic acid is oxidized to 4-nitrobenzenesulfonic acid anion, which is ultimately broken down through the number of steps of the reactions into CO_2_ and H_2_O [50,51]. 

## 3. Experimental Section

### 3.1. Materials

Potassium iodide (KI), bismuth (III) nitrate pentahydrate [Bi(NO_3_)_3_·5H_2_O] and methylene blue (MB) were acquired from WarChem Sp z o.o. (Warsaw, Poland) and utilized for the chemical reaction without any additional purification. The analytical-grade nitric acid (HNO_3_), obtained from Merck in Darmstadt, Germany, was used for the chemical reaction. The deionized water and high-analytical-grade ethanol [99.0%, WarChem Sp z o.o. (Warsaw, Poland)] were used for purification and chemical reactions.

### 3.2. Synthesis of Bismuth Triiodied (BiI_3_) Microplates 

The synthesis of BiI_3_ powder was conducted through the solution-mixing method. The preparation procedure was as follows: First, the stock solutions were prepared by dissolving 0.081 mmol of Bi (NO_3_)_3_ and 5H_2_O in 1 mL of HNO_3_ solution with vigorous stirring for 10 min, which was made up to 10 mL using deionized water. Thereafter, 0.49 mmol KI was dissolved in 10 mL water. Subsequently, 1:5 molar concentration of these two precursor solutions was taken in a 1:1 volume ratio to make up 10 mL of total volume. The reaction mixture was stirred for 4 h. After the completion of the reaction, the final black precipitate was washed with deionized water repeatedly to remove any residual trace of reactants. The entire reaction was carried out at ambient temperature while the final product dried at 60 °C for 30 min in a vacuum oven.

### 3.3. Conversion of Bismuth Triiodied (BiI_3_) Microplates into Bismuth Oxyiodide (BiOI) Nanoflakes through Ultrasonication

**Figure 9:**
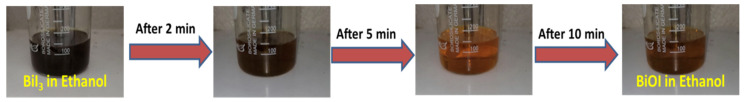
The digital image of the solution color changes during the conversion process at different time intervals.

The BiI_3_ microplates were converted into BiOI nanoflakes through an ultrasonication process. For this ultrasonic conversion process, the cylinder in T-horn ultrasonic reactor [750 Watt ultrasonic processor VCX-750 with a sealed VC-334 converter (Sonics & Materials, Inc., Newtown, CT, USA)] was partially immersed in the reaction mixture. For 10–15 min, the reaction mixture was exposed to ultrasonic radiation at a frequency of 20 kHz and a power density of 118.4 W/cm^2^. The conversion process was as follows: First, 200 mg of previously synthesized was added into 100 ml of ethanol. After that, the black color mixture was inserted into a sonicator and sonicated for 10 min. During sonication, the black mixture was gradually converted into red, which indicates the conversion of BiI_3_ microplates into BiOI plates. The conversion process was completed within 10 min, which was indicated by the complete change in the color of the reaction mixture from black to red. Later, the red precipitate was washed with deionized water and ethanol repeatedly to remove any residual trace of reactants. Finally, the final product was dried at 60 °C overnight in a vacuum oven. The gradual change in the solution color during ultrasonication at different time intervals is presented in Figure 9. Also, the conversion process is presented in the Supplementary Video S1 (the video has been sped up by 6×).

### 3.4. Characterization Techniques

The morphology and elemental composition of both the synthesized BiI_3_ and the converted BiOI were comprehensively examined using scanning electron microscopy (SEM) and energy-dispersive X-ray spectroscopy (EDX). These analyses were conducted by employing the Phenom Pro X microscope from Phenom World (Eindhoven, Netherlands), which is equipped with an integrated EDX spectrometer. The obtained EDX spectra were carefully analyzed using the ProSuite Element Identification software from Phenom-World. The microstructure analysis of the samples was conducted by utilizing the JEOL JEM-3010 high-resolution transmission electron microscope (TEM) manufactured by JEOL Ltd. Tokyo, Japan. The microscope operated at an acceleration voltage of 300 kV and was outfitted with a Gatan 2 k × 2 k Orius^TM^ 833 SC200D CCD camera from Gatan Inc. based in Pleasanton, CA, USA. To prepare the samples for analysis, the powder samples were dispersed in isopropano and the resulting material was subsequently deposited onto a Cu grid featuring an amorphous carbon film specifically designed for TEM observations. XRD studies were carried out using the D8 Advance diffractometer manufactured by Bruker (Karlsruhe, Germany), employing a Cu-Kα cathode (λ = 1.54 Å) operating at a voltage of 40 kV and a current of 40 mA. A scanning step of 0.02° with a scan rate of 0.40°/min within the angle (2Θ) range from 10° to 80° was employed for data acquisition. The International Centre for Diffraction Data (ICDD) PDF#2 database (Bismuth Triiodide (PCPDF-48-1795), Bismuth Oxyiodide (PDF Number 04-012-5693), access date: 1 September 2024) was utilized to analyze and identify the phases present in the XRD spectrum. The diffuse reflectance spectroscopy (DRS) spectrum was used to monitor the ultrasonic conversion process at room temperature utilizing the PC-2000 spectrophotometer manufactured by Ocean Optics Inc. (Dunedin, FL, USA). This spectrophotometer was connected to the ISP-REF integrating sphere, also from Ocean Optics Inc.

### 3.5. Photocatalytic Activity Test

The photocatalytic efficiency of the ultrasonically converted BiOI was tested by the degradation of methylene blue (MB) (20 mL, 30 mg/ liter, pH = 6.8) in the aqueous medium in the presence of UV light. The photolysis investigations were carried out by irradiating the MB solution with UV lamps [(Sineo, Shanghai, China), λ = 365 nm, power of 300 W]. Before the irradiation of MB, the system was held in darkness for an hour to achieve an adsorption–desorption equilibrium between BiOI and MB. The variations in the UV-vis absorption spectra of irradiated aqueous MB solutions over time were monitored using a PC2000 spectrophotometer, DH2000-FHS lamp and the OOI-Base software (version 1.5, Ocean Optics, Inc. Dunedin, FL, USA). The manufacturer of that equipment was Ocean Optics Inc. (Dunedin, FL, USA). For such measurements, 1 mL aliquots were withdrawn from the reaction mixture initially (t = 0) and after different interval times (t) of UV illumination. Also, to study the effect of the loading of BiOI as a catalyst on the degradation of MB, we performed the catalytic experiment with 5 and 10 mg of BiOI sample. Based on the spectra, the rate of MB degradation and degradation efficiency were determined, which are discussed later. Furthermore, the reusability of BiOI nanoflakes was performed with the 10 mg sample under above mention similar condition. After each degradation cycle, the catalyst was recovered, washed and reused under identical conditions.

## 4. Conclusions

In summary, this study successfully demonstrated the ultrasonic-assisted rapid and facile conversion of micrometer-sized BiI_3_ particles into BiOI nanoflakes, showcasing a promising route for the synthesis of advanced 2D nanomaterials. The mechanism underlying the ultrasonic-assisted conversion involves several key factors. The ultrasonic waves generate cavitation bubbles in the reaction medium, leading to localized heating, intense shear forces and microstreaming. These phenomena promote the breakdown of precursor particles and facilitate chemical reactions by enhancing mass transport and accelerating reaction kinetics. Additionally, the sonochemical effects induce the formation of highly reactive species, which further contribute to the conversion process. The characterization of the synthesized BiOI nanoflakes confirmed their uniform morphology, crystalline structure and high surface area, attributes crucial for photocatalytic applications. Indeed, the BiOI nanoflakes exhibited superior photocatalytic activity towards MB compared to conventional catalysts under UV light irradiation. This enhanced performance can be attributed to several factors inherent to the BiOI nanostructure. Firstly, the large surface area and high surface-to-volume ratio of the nanoflakes facilitate the efficient adsorption of target molecules, promoting their degradation. Secondly, the unique electronic structure of BiOI enables the effective utilization of visible light photons, extending the range of wavelengths for photocatalytic activity. Moreover, the ultrasonic-assisted synthesis process likely introduced defects and vacancies in the BiOI lattice, which serve as active sites for charge separation and catalytic reactions. Overall, the findings of this study highlight the potential of ultrasonic-assisted methods for the fabrication of advanced photocatalysts and underscore the importance of nanostructuring in enhancing photocatalytic performance. The insights gained contribute to the development of sustainable technologies for environmental remediation and wastewater treatment, addressing pressing challenges in pollution control and resource conservation. Further research in this direction holds promise for the design and synthesis of tailored 2D nanomaterials with optimized properties for diverse photocatalytic applications.

## Figures and Tables

**Table 1:** Comparison of the degradation rate of MB with previously published results.

Name of the catalysts	Performance	Source of Light	References
1% Fe-doped In_2_O_3_	83% degradation in 360 min	UV-Irradiation	[41]
Fe_2_O_3_–TiO_2_ composite	70% degradation in 150 min	Xe lamp (500 W)	[42]
SnO_2_ nanoparticles calcined at 300 °C	51.3% degradation in 180 min	UV-Irradiation	[43]
Hierarchical Cu_2_O nanocubes	55% degradation in 120 min	200 W Hg–Xe arc lamp	[44]
ZnO	67.78% degradation in 60 min	UV-Irradiation	[45]
HNbWO_6_ nanosheets	62.50% degradation in 360 min	UV-Irradiation	[46]
Poly(TMPT)/TiO_2_	51.50% degradation in 420 min	UV-Irradiation	[47]
ZrO_2_	33.0% degradation in 120 min	UV-Irradiation	[48]
BiOI	90% degradation in 480 min	UV-Irradiation	Our work

## Data Availability

Data will be made available on request.

## Supplementary Materials

The following supporting information can be downloaded at https://www.mdpi.com/article/10.3390/ijms251910265/s1.

## References

[1] Wagner B., Huttner A., Bischof D., Engel A., Witte G., Heine J. (2020). Chemical surface reactivity and morphological changes of bismuth triiodide (BiI_3_) under different environmental conditions. Langmuir.

[2] Zavabeti A., Jannat A., Zhong L., Haidry A.A., Yao Z., Ou J.Z. (2020). Two-Dimensional Materials in Large-Areas: Synthesis, Properties and Applications. Nano-Micro Lett..

[3] Abdelhamid H.N. (2024). Nanocellulose-Based Materials for Water Pollutant Removal: A Review. Int. J. Mol. Sci..

[4] Wang L., Hu P., Long Y., Liu Z., He X. (2017). Recent advances in ternary two-dimensional materials: Synthesis, properties and applications. J. Mater. Chem. A.

[5] Hong J., Chu Z., Li C., Yang W., Kawi S., Ye Q. (2024). Innovative Bi_5_O_7_I/MIL-101 (Cr) Compounds: A Leap Forward in Photocatalytic Tetracycline Removal. Int. J. Mol. Sci..

[6] Ganose A.M., Cuff M., Butler K.T., Walsh A., Scanlon D.O. (2016). Interplay of orbital and relativistic effects in bismuth oxyhalides: BiOF, BiOCl, BiOBr, and BiOI. Chem. Mater..

[7] Arumugam M., Choi M.Y. (2019). Recent progress on bismuth oxyiodide (BiOI) photocatalyst for environmental remediation. J. Ind. Eng. Chem..

[8] Meng L., Jian J., Yang D., Dan Y., Sun W., Ai Q., Zhang Y., Zhou H. (2024). Hydrophilicity and Pore Structure Enhancement in Polyurethane/Silk Protein–Bismuth Halide Oxide Composite Films for Photocatalytic Degradation of Dye. Int. J. Mol. Sci..

[9] Ye L., Tian L., Peng T., Zan L. (2011). Synthesis of highly symmetrical BiOI single-crystal nanosheets and their {001} facet-dependent photoactivity. J. Mater. Chem..

[10] Matiur R.M., Noman M., Kato S., Soga T. (2021). A novel modest synthesis of device applicable flakes based stable BiOI film by the oxidation of BiI_3_ film. J. Alloys Compd..

[11] Huang H., Liu K., Zhang Y., Chen K., Zhang Y., Tian N. (2014). Tunable 3D hierarchical graphene–BiOI nanoarchitectures: Their in situ preparation, and highly improved photocatalytic performance and photoelectrochemical properties under visible light irradiation. RSC Adv..

[12] Zhang K.-L., Liu C.-M., Huang F.-Q., Zheng C., Wang W.-D. (2006). Study of the electronic structure and photocatalytic activity of the BiOCl photocatalyst. Appl. Catal. B Environ..

[13] Di J., Xia J., Li H., Guo S., Dai S. (2017). Bismuth oxyhalide layered materials for energy and environmental applications. Nano Energy.

[14] Sun Z., Amrillah T. (2024). Potential application of bismuth oxyiodide (BiOI) when it meets light. Nanoscale.

[15] Zhou L., Wang W., Zhang L. (2007). Ultrasonic-assisted synthesis of visible-light-induced Bi_2_MO_6_ (M = W, Mo) photocatalysts. J. Mol. Catal. A Chem..

[16] An C., Wang T., Wang S., Chen X., Han X., Wu S., Deng Q., Zhao L., Hu N. (2023). Ultrasonic-assisted preparation of two-dimensional materials for electrocatalysts. Ultrason. Sonochem..

[17] Gedanken A. (2004). Using sonochemistry for the fabrication of nanomaterials. Ultrason. Sonochem..

[18] Li Z., Dong J., Zhang H., Zhang Y., Wang H., Cui X., Wang Z. (2021). Sonochemical catalysis as a unique strategy for the fabrication of nano-/micro-structured inorganics. Nanoscale Adv..

[19] Foroughi F., Lamb J.J., Burheim O.S., Pollet B.G. (2021). Sonochemical and Sonoelectrochemical Production of Energy Materials. Catalysts.

[20] Gaudino E.C., Cravotto G., Manzoli M., Tabasso S. (2020). Sono- and mechanochemical technologies in the catalytic conversion of biomass. Chem. Soc. Rev..

[21] Song G., Ma S., Tang G., Wang X. (2010). Ultrasonic-assisted synthesis of hydrophobic magnesium hydroxide nanoparticles. Colloids Surf. A Physicochem. Eng. Asp..

[22] Xu H., Zeiger B.W., Suslick K.S. (2013). Sonochemical synthesis of nanomaterials. Chem. Soc. Rev..

[23] Frecentese F., Sodano F., Corvino A., Schiano M.E., Magli E., Albrizio S., Sparaco R., Andreozzi G., Nieddu M., Rimoli M.G. (2023). The Application of Microwaves, Ultrasounds, and Their Combination in the Synthesis of Nitrogen-Containing Bicyclic Heterocycles. Int. J. Mol. Sci..

[24] Crovetto A., Hajijafarassar A., Hansen O., Seger B., Chorkendorff I., Vesborg P.C. (2020). Parallel evaluation of the BiI_3_, BiOI, and Ag_3_BiI_6_ layered photoabsorbers. Chem. Mater..

[25] Matiur R.M., Abuelwafa A.A., Putri A.A., Kato S., Kishi N., Soga T. (2021). Annealing effects on structural and photovoltaic properties of the dip-SILAR-prepared bismuth oxyhalides (BiOI, Bi_7_O_9_I_3_, Bi_5_O_7_I) films. SN Appl. Sci..

[26] Sun H., Yang D., Liu Y., Zhu X. (2019). Highly Flexible X-ray Detectors Based on Pure Inorganic Metal Iodide Polycrystalline Thin Films as Photon-to-Charge Conversion Layers. ACS Appl. Electron. Mater..

[27] Hojamberdiev M., Vargas R., Madriz L., Yubuta K., Kadirova Z.C., Shaislamov U., Sannegowda L.K., Jędruchniewicz K., Typek R., Teshima K. (2023). Unveiling the origin of the efficient photocatalytic degradation of nitazoxanide over bismuth (oxy)iodide crystalline phases. Environ. Sci. Nano.

[28] Prasad M.D., Sangani L.D.V., Batabyal S.K., Krishna M.G. (2018). Single and twinned plates of 2D layered BiI_3_ for use as nanoscale pressure sensors. CrystEngComm.

[29] Wilczewska P., Bielicka-Giełdoń A., Szczodrowski K., Malankowska A., Ryl J., Tabaka K., Siedlecka E.M. (2021). Morphology regulation mechanism and enhancement of photocatalytic performance of BiOX (X = Cl, Br, I) via mannitol-assisted synthesis. Catalysts.

[30] Patterson A.L. (1939). The Scherrer Formula for X-ray Particle Size Determination. Phys. Rev. B.

[31] Nowak M., Kauch B., Szperlich P. (2009). Determination of energy band gap of nanocrystalline SbSI using diffuse reflectance spectroscopy. Rev. Sci. Instrum..

[32] Hung P.T., Hien V.X., Hoat P.D., Lee S., Lee J.-H., Kim J.-J., Heo Y.-W. (2019). Photo induced NO_2_ sensing properties of bismuth triiodide (BiI_3_) nanoplates at room temperature. Scr. Mater..

[33] Madelung O. (2000). Ternary Compounds, Organic Semiconductors. Landolt-Börnstein—Group III Condensed Matter. https://materials.springer.com/bp/docs/978-3-540-31362-5.

[34] Dheyab M.A., Aziz A.A., Jameel M.S., Khaniabadi P.M., Mehrdel B. (2019). Mechanisms of effective gold shell on Fe_3_O_4_ core nanoparticles formation using sonochemistry method. Ultrason. Sonochemistry.

[35] Altay R., Sadaghiani A.K., Sevgen M.I., Şişman A., Koşar A. (2020). Numerical and Experimental Studies on the Effect of Surface Roughness and Ultrasonic Frequency on Bubble Dynamics in Acoustic Cavitation. Energies.

[36] Ehsani M., Zhu N., Doan H., Lohi A., Abdelrasoul A. (2021). In-situ synchrotron X-ray imaging of ultrasound (US)-generated bubbles: Influence of US frequency on microbubble cavitation for membrane fouling remediation. Ultrason. Sonochem..

[37] Alshehri A.A., Malik M.A. (2019). Biogenic fabrication of ZnO nanoparticles using Trigonella foenum-graecum (Fenugreek) for proficient photocatalytic degradation of methylene blue under UV irradiation. J. Mater. Sci. Mater. Electron..

[38] Mohamed M.M., Al-Esaimi M.M. (2006). Characterization, adsorption and photocatalytic activity of vanadium-doped TiO_2_ and sulfated TiO_2_ (rutile) catalysts: Degradation of methylene blue dye. J. Mol. Catal. A Chem..

[39] Nolan N.T., Synnott D.W., Seery M.K., Hinder S.J., Van Wassenhoven A., Pillai S.C. (2011). Effect of N-doping on the photocatalytic activity of sol–gel TiO_2_. J. Hazard. Mater..

[40] Mistewicz K., Kępińska M., Nowak M., Sasiela A., Zubko M., Stróż D. (2020). Fast and Efficient Piezo/Photocatalytic Removal of Methyl Orange Using SbSI Nanowires. Materials.

[41] Jabeen S., Iqbal J., Arshad A., Awan M., Warsi M. (2019). (In_1−x_Fe_x_)_2_O_3_ nanostructures for photocatalytic degradation of various dyes. Mater. Chem. Phys..

[42] Jo W.-K., Selvam N.C.S. (2015). Synthesis of GO supported Fe_2_O_3_–TiO_2_ nanocomposites for enhanced visible-light photocatalytic applications. Dalton Trans..

[43] Sadeghzadeh-Attar A. (2018). Efficient photocatalytic degradation of methylene blue dye by SnO_2_ nanotubes synthesized at different calcination temperatures. Sol. Energy Mater. Sol. Cells.

[44] Kumar S., Parlett C.M., Isaacs M.A., Jowett D.V., Douthwaite R.E., Cockett M.C., Lee A.F. (2016). Facile synthesis of hierarchical Cu2O nanocubes as visible light photocatalysts. Appl. Catal. B Environ..

[45] Lin J., Luo Z., Liu J., Li P. (2018). Photocatalytic degradation of methylene blue in aqueous solution by using ZnO-SnO_2_ nanocomposites. Mater. Sci. Semicond. Proc..

[46] Hu L.-F., Li R., He J., Da L.-G., Lv W., Hu J.-S. (2015). Structure and photocatalytic performance of layered HNbWO_6_ nanosheet aggregation. J. Nanophotonics.

[47] Jamal R., Osman Y., Rahman A., Ali A., Zhang Y., Abdiryim T. (2014). Solid-State Synthesis and Photocatalytic Activity of Polyterthiophene Derivatives/TiO_2_ Nanocomposites. Materials.

[48] Khaksar M., Amini M., Boghaei D.M., Chae K.H., Gautam S. (2015). Mn-doped ZrO_2_ nanoparticles as an efficient catalyst for green oxidative degradation of methylene blue. Catal. Commun..

[49] Kulis-Kapuscinska A., Kwoka M., Borysiewicz M.A., Wojciechowski T., Licciardello N., Sgarzi M., Cuniberti G. (2023). Photocatalytic degradation of methylene blue at nanostructured ZnO thin films. Nanotechnology.

[50] Wang X.-Q., Han S.-F., Zhang Q.-W., Zhang N., Zhao D.-D. (2018). Photocatalytic oxidation degradation mechanism study of methylene blue dye waste water with GR/iTO_2_. MATEC Web Conf..

[51] Huang F., Chen L., Wang H., Yan Z. (2010). Analysis of the degradation mechanism of methylene blue by atmospheric pressure dielectric barrier discharge plasma. Chem. Eng. J..

