# Supplementary material for: Ultrasonic-Assisted Conversion of Micrometer-Sized BiI3 into BiOI Nanoflakes for Photocatalytic Applications
Source: Int J Mol Sci. 2024 Sep 24;25(19):10265. doi: 10.3390/ijms251910265 (PMC11476912; doi:10.3390/ijms251910265)
Supplement: Supplementary file 1 [file ijms-25-10265-s001.zip › Supporting Informations.pdf]

Supporting Information

Ultrasonic Assisted Conversion of Micrometer Size BiI_3 into BiOI Nanoflakes for their Photocatalytic Applications

Tushar Kanti Das [1,*], Marcin Jesionek [1,*], Krystian Mistewicz [1], Bartłomiej Nowacki [2], Mirosława Kępińska [1], Maciej Zubko [3,4], Marcin Godzierz [5], Anna Gawron [5]

[1]Institute of Physics – Center for Science and Education, Silesian University of Technology, Krasińskiego 8, 40-019 Katowice, Poland

[2]Department of Industrial Informatics, Faculty of Materials Science, Joint Doctorate School, Silesian University of Technology, Krasinskiego 8, 40-019, Katowice, Poland

[3]Institute of Materials Engineering, Faculty of Science and Technology, University of Silesia, 75 Pułku Piechoty 1a, 41-500 Chorzów, Poland

[4]Department of Physics, Faculty of Science, University of Hradec Králové, Rokitanského 62, 500 03, Hradec Králové, Czech Republic

[5]Centre of Polymer and Carbon Materials, Polish Academy of Sciences, 41-819 Zabrze, Poland

* Correspondence: tushar.kanti.Das@polsl.pl (T.K.D.); Marcin.Jesionek@polsl.pl (M.J.)

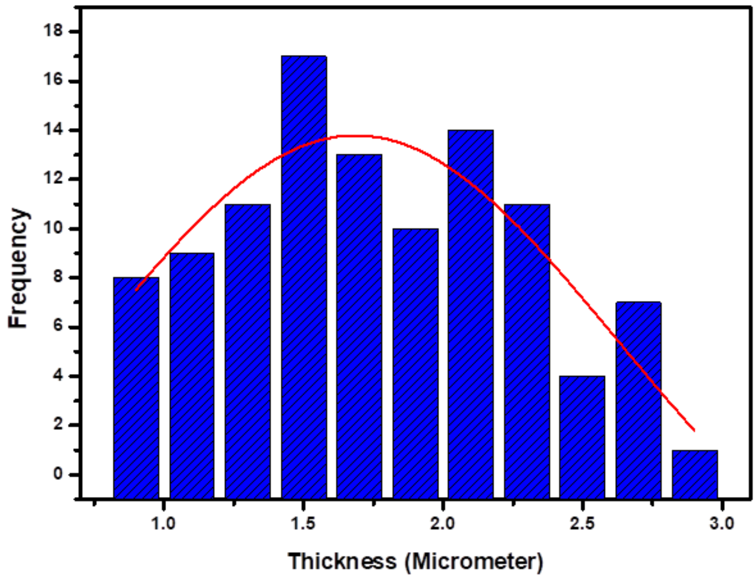

Figure S1 Histograms of the distribution of BiI_3 microplates thickness (The red line represents Gaussian fits to the respective histogram data).

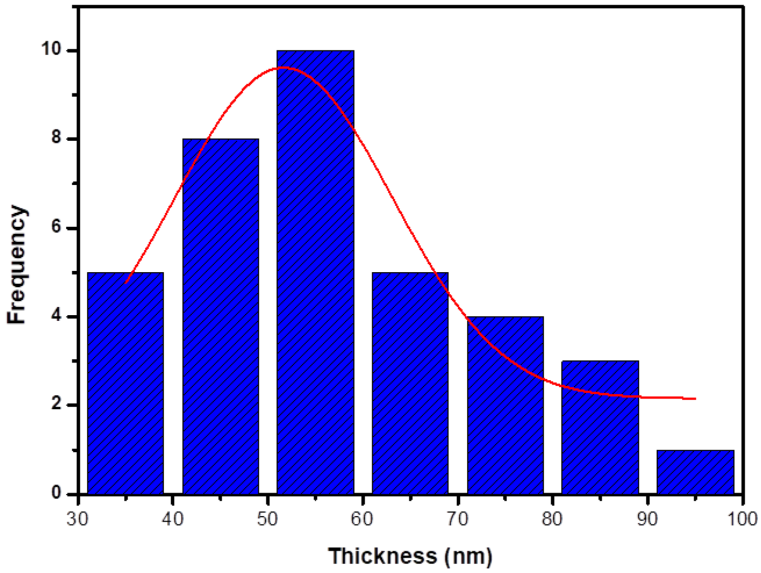

Figure S2 Histograms of the distribution of BiOI nanoflakes thickness (The red line represents Gaussian fits to the respective histogram data).

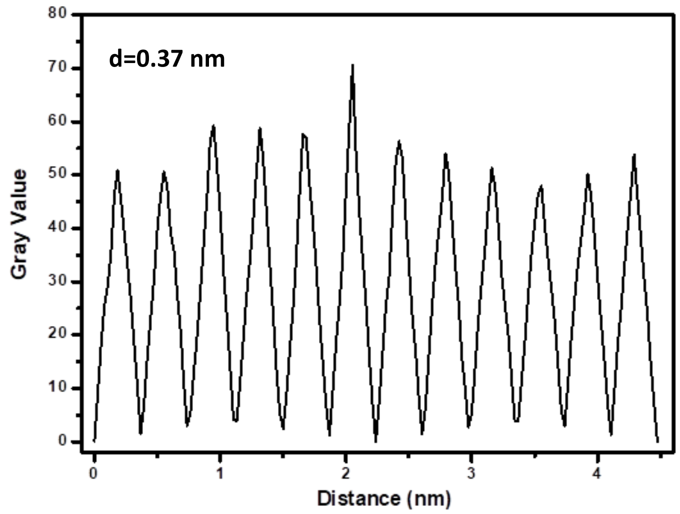

Figure S3 Gray scale plot profile measured from high resolution TEM images of BiI_3 **(Figure 2(c))** using IMAGE J software.

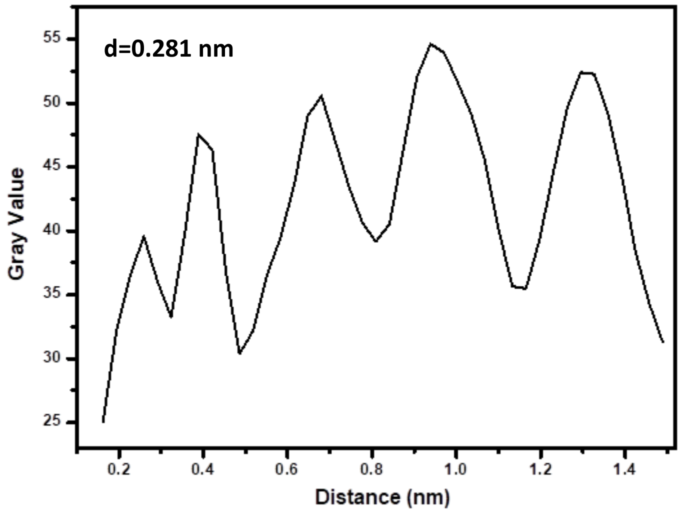

Figure S4 Gray scale plot profile measured from high resolution TEM images of BiOI **(Figure 2(f))** using IMAGE J software.
